# The Potential Association Between Periodontal Diseases and Adverse Pregnancy Outcomes in Pregnant Women: A Systematic Review of Randomized Clinical Trials

**DOI:** 10.7759/cureus.33216

**Published:** 2023-01-01

**Authors:** Bashayer H Alnasser, Njoud K Alkhaldi, Waad K Alghamdi, Faisal T Alghamdi

**Affiliations:** 1 Obstetrics and Gynecology, Maternity and Children Hospital, Dammam, SAU; 2 General Medicine and Surgery, College of Medicine, Princess Nourah Bint Abdulrahman University, Riyadh, SAU; 3 Oral Biology, Faculty of Dentistry, King Abdulaziz University, Jeddah, SAU

**Keywords:** review, randomized clinical trial, pregnancy outcome, pregnant women, periodontitis, association

## Abstract

Preceding studies have demonstrated that periodontitis might increase the liability of adverse pregnancy outcomes such as preterm birth, preeclampsia, low birth weight, and perinatal fatality in pregnant women. Nonetheless, there is no convincing testimony that periodontitis is related directly to adverse pregnancy outcomes in pregnant women. This systematic review intended to assess and review all the available randomized clinical trials that concentrated on the association between periodontal diseases and adverse pregnancy outcomes, and the impact of periodontal disease therapy on adverse pregnancy outcomes. The databases like Scopus, PubMed, Google Scholar, and Web of Science were consumed to explore relevant and suitable studies after adopting the inclusion and exclusion criteria. The search included articles with no time restrictions and certain keywords were utilized in the databases. The investigation was done through four independent reviewers employing the Preferred Reporting Items for Systematic Reviews and Meta-Analyses (PRISMA) guidelines. Twenty-three studies fulfilled the exclusion and inclusion criteria and were used and included in this systematic review. The above-mentioned studies assessed the association between periodontal diseases and adverse pregnancy outcomes and the effect of periodontal disease treatment in reducing the influence of adverse pregnancy outcomes. This systematic review revealed that there is a relationship between periodontitis and adverse pregnancy outcomes, and periodontal treatment has a reducing impact on adverse pregnancy outcomes in pregnant women with periodontitis. Prospect studies are warranted to investigate the relationship between periodontitis and different adverse pregnancy outcomes and to decide the best type and the most effective therapy to treat periodontitis in pregnant women.

## Introduction and background

Periodontitis and consecutive immunological reactions are hypothesized to cause adverse pregnancy outcomes such as low birth weight, preeclampsia, preterm birth, gestational diabetes, and perinatal fatality [1]. Periodontal disease, such as periodontitis and gingivitis, is an infection-caused ailment and is an immuno-inflammatory reaction disturbing the tissues promoting the teeth (gingiva, periodontal ligaments, alveolar bone, and cementum) [2]. Periodontitis is a comparatively prevalent ailment, which appears in more than 30% of humans in several communities [3]; the prevalence is between 5% and 20% in pregnant women [4]. A study presumed that the change in progesterone and estrogen levels through pregnancy applies the impact on inflammatory responses and subgingival microbiota in gingival tissues through the changes of chemotaxis, enzymes, cytokines, and antioxidants from gingival fibroblasts (GFs), polymorphonuclear leukocytes (PMNs), and periodontal ligament cells (PDLCs). These changes indirectly commit to increased gingival inflammation. Although, the mechanisms responsible for these changes are not fully known [5]. Moreover, investigators have proposed that periodontal disease leads to the release of inflammatory mediators such as cytokines or pathogens, which then impact amniotic fluid or embryonic tissue through hematogenous transport [4].

In 1996, Offenbacher was the first researcher who chronicled and proposed that pregnant women diagnosed to have periodontitis have a greater risk to deliver preterm and low birth weight infants in comparison with healthy periodontal tissue by 7.5 times [2]. Subsequently, many studies conducted and documented a rapport between periodontitis and adverse pregnancy outcomes, such as Meqa et al. [6], an investigator who reported a significant correlation between periodontitis and low birth weight as well as preterm birth. The adjusted odds were 3.2 times greater for women diagnosed to have periodontitis to have a low-weighted generation in comparison to women without periodontitis. Also, it is 3.4 times greater for women diagnosed to have periodontitis to give premature birth in comparison to women without periodontitis. On the other hand, a prospective study done by Farrell et al. documented no association between periodontal condition and either preterm birth or low birth weight in non-smoker pregnant women in the first three months of the pregnancy. However, in subjects with no previous history of smoking, there was a weak but statistically significant relationship between poor periodontal condition and late abortion [7].

Therefore, it remains unclear if periodontal disease is related to and leads to adverse pregnancy outcomes and if treatment during pregnancy is influential to preclude it. Few reviews have discussed the relationship between periodontal diseases and adverse pregnancy outcomes [1,8-15]. Thus, this systematic review intended to evaluate and review all the available randomized clinical trials that focused on the relationship between periodontal diseases and adverse pregnancy outcomes and the impact of periodontal disease treatment on adverse pregnancy outcomes.

## Review

Materials and methods

Four independent reviewers accomplished this literature review following the Preferred Reporting Items for Systematic Reviews and Meta-Analyses (PRISMA) guidelines [16].

Focus Review Question

The review question is as follows: “Can periodontal therapy be used as a reducing factor of adverse pregnancy outcomes to treat pregnant women with periodontitis?”

Information Sources

PubMed, Google Scholar, Web of Science, and Scopus were utilized for an electronic exploration of studies in the English language with no time limits because of the inadequacy of updated systematic reviews that were insured in the dental and obstetric field.

Literature Search Strategy

In July 2022, a literature exploration through PubMed, Google Scholar, Web of Science, and Scopus was done. The search utilized the following specific keywords: “periodontitis,” “periodontal,” “pre-eclampsia,” “periodontal disease,” “periodontal treatment,” “preeclampsia,” “pregnancy outcomes,” “pregnancy complications,” “pregnancy,” “preterm,” “premature birth,” “overweight pregnancy,” “oral health,” “oral microbe,” ”gestational diabetes,” “maternal periodontitis,” and “low birth weight.”

Inclusion Criteria

Studies that were conducted on human subjects only and published in the English language were involved in this systematic review. All articles included in the systematic review were randomized control clinical trials with no time restriction.

Exclusion Criteria

Articles were rejected if they met any of the following criteria: observation studies, review articles, observational or cohort studies, case reports, personal opinion articles, in vitro/in vivo studies or editorial, non-English language studies, and articles that discussed periodontitis and its therapy in non-pregnant women, articles that did not mention any information related to the adverse pregnancy outcome in case of usage of periodontal therapy, and articles that were conducted on nonhuman sources.

Critical Appraisal

The reviewers independently evaluated the titles and abstracts of the papers based on the eligibility criteria. All disagreements between the four reviewers were resolved through consensus and discussion.

Data Extraction

Data were extracted after comprehensively reading the studies and considering the following variables: title, abstract, methods, and the association of periodontal therapy with adverse pregnancy outcomes. All reviewers independently verified the data for completeness and accuracy before entering the data into standardized Microsoft Excel spreadsheets (Microsoft Corporation, Redmond, WA).

Data Items

Data from the chosen articles were accumulated and classified into columns based on the following information: author, country, year, number of participants, patient’s age, periodontal status, gestational status, types of adverse pregnancy outcomes, and the main outcomes of the study.

Methodological Quality and Risk of Bias Assessment of Included Studies

The methodological quality of each article was accomplished utilizing the risk of bias assessment tool outlined in the Cochrane risk-of-bias visualization (robvis) [17]. The Cochrane Collaboration advocates a specific tool to assess the risk of bias in each selected article. The four authors investigated the risk of bias in the included articles based on the following seven domains: random sequence generation, allocation concealment, blinding of participants and personnel, blinding of outcome assessment, incomplete outcome data, selective reporting, and other sources of bias. Each domain was investigated as "low," "unclear," or "high." These assessments were stated for each included article in the "risk of bias" figures. The overall risk of bias associated with each article was graded as follows: low risk of bias: all domains were assessed as "low risk"; unclear risk of bias: at least one domain was assessed as "unclear risk"; and high risk of bias: at least one domain was assessed as "high risk." The risk of bias was investigated during the course of data extraction, which could impress the outcome of each included article.

Types of Outcome Measurements

Primary outcomes: The number of articles reported significant and non-significant associations between periodontal diseases and adverse pregnancy outcomes.

Secondary outcomes: The effect of periodontal diseases on adverse pregnancy outcomes such as low birth weight, preterm birth, preeclampsia, eclampsia, HELLP (hemolysis, elevated liver enzymes, and low platelet count) syndrome, intrauterine growth restriction, and gestational diabetes. In addition, several articles reported significant and non-significant associations between periodontal disease treatment and reducing adverse pregnancy outcomes.

Synthesis of Results

Only one table was arranged to gather the included study’s characteristics. It also includes the main outcomes of the studies that studied the relationship between periodontal disease and adverse pregnancy outcomes.

Statistician Analysis

Meta-analysis was impossible because of the discrepancy in the included articles. Parametric data relating to the patient’s age in the included articles are presented as mean and standard deviation (mean ± SD), in addition to a descriptive evaluation of the findings.

Results

Study Selection

**Figure 1:**
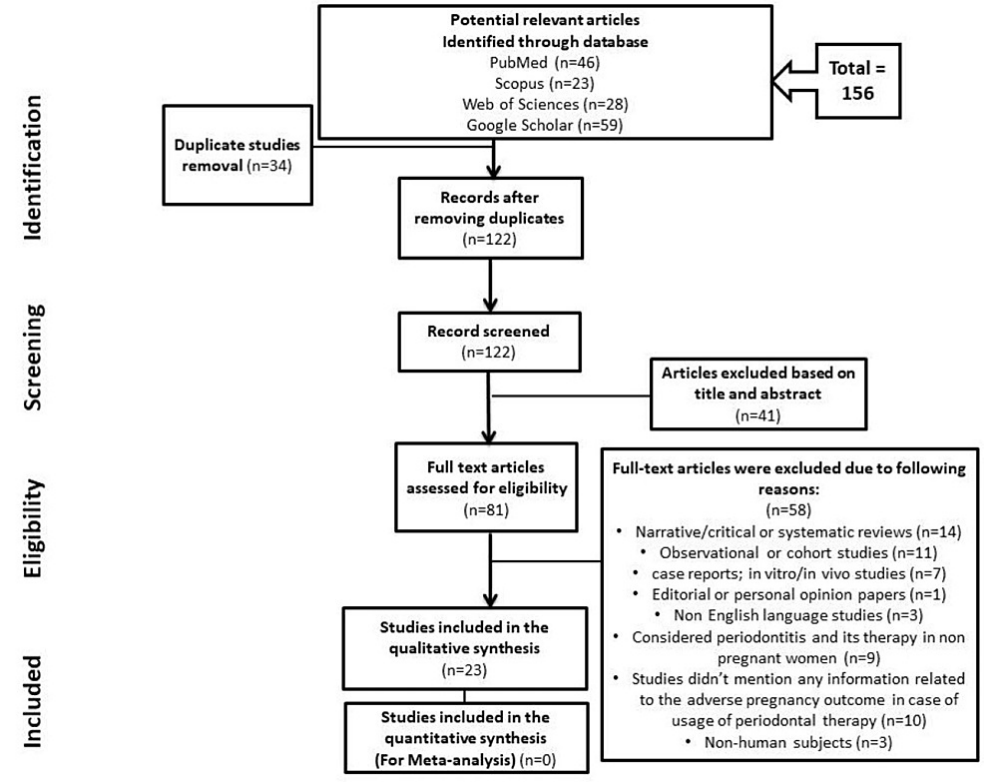
PRISMA flow chart of study selection PRISMA: Preferred Reporting Items for Systematic Reviews and Meta-Analyses.

In the beginning, keywords were utilized to have a total of 156 studies from databases. Thirty-four studies were rejected because of their irrelevance or duplicity. Only 122 studies were screened. Forty-one studies were rejected and excluded based on title and abstract. Following assessment for eligibility, after excluding 58 studies due to different reasons, only 23 studies were included in this systematic review. Figure 1 displays the search flow chart summary for this systematic review.

Study Characteristics

This systematic review included 23 human articles that met the inclusion criteria. These studies investigated the relationship between periodontitis and adverse pregnancy outcomes, as well as the effectiveness of periodontal treatment as a factor in reducing adverse pregnancy outcomes in pregnant women with periodontal diseases [3,6,18-38]. Only randomized control clinical trials were included in the systematic review, with a total sample size of 9724 participants; the studies were conducted in 12 countries, including the United States, Ireland, Hungary, Spain, Republic of Kosovo, Australia, China, India, Iran, Brazil, Colombia, and Chile (Table 1). Even though there were no time constraints in this systematic review, all included studies were published from 2002 to 2019. The participants' ages ranged from 16 to 43 years. Eight studies reported the patient’s age within a range and a mean age with standard deviation (mean ± SD) [18,26,27,31,35-38], three studies reported the patient's age within a range only [6,22,25], three studies reported the minimum patient's age [20,21,32], seven studies reported a minimum age within a range and a mean age with standard deviation (mean ± SD) [19,23,24,28-30,34], and two studies reported the mean age with standard deviation only [3,33]. Caneiro-Queija et al. [18] reported that the ages of subjects ranged from 18 to 40 years old, with a mean age and standard deviation in the control group of 32.25 ± 4.21 and 32.14 ± 4.27 in the test group, whereas Weidlich et al. [25] reported that the ages ranged from 18 to 35 years old. Jiang et al. [21,23] disclosed that the subjects were at least 18 years old, whereas Michalowicz et al. [34] reported that the subjects were at least 16 years old, with a mean age and standard deviation of 25.9 ± 5.5 in the control group and 26.1 ± 5.6 in the treatment group. Furthermore, Novak et al. [33] reported that the control group's mean age was 25.7, while in the treatment group was 27.3. Furthermore, all of the studies included structured their inclusion and exclusion criteria based on the participants' periodontal, medical, and gestational status. To screen and examine the oral cavity for periodontal diseases, various criteria and methods were used. Some studies defined periodontitis as attachment loss of 3 mm on three teeth [3,29]. The gestational age was specified in almost all of the included studies, but it varied from one to the next. Pregnant women in their first trimester were included in some studies [18,19,34], while pregnant women in their third trimester were included in one study [23]. Some articles, on the other hand, included all pregnant women, regardless of gestational age [29,33]. Furthermore, one study limited its population to pregnant women with preeclampsia [30], while another included non-pregnant women planning to conceive within the next year [24]. Only singleton pregnancy was mentioned in 10 articles [19,25,27,31-35,37,38]. Various adverse pregnancy outcomes were observed and reported in the included studies. Almost all of them mentioned preterm birth and low birth weight as negative pregnancy outcomes. Furthermore, some studies found abortion [19,27], stillbirth [19,20,32], gestational diabetes and hypertension [21], and preeclampsia and HELLP syndrome [30]. Table 1 depicts an informative summary of the article's features.

**Table 1:** Study characteristics of included studies. HELLP: hemolysis, elevated liver enzymes, and low platelet count.

Authors, country	Year	Number of participants/age of patients (mean ± SD)	Periodontal status	Gestational status	Types of adverse outcomes of pregnancy	Main outcomes of the study
Macones et al. [3], United States	2010	n = 757. Control group (24.4 ± 5.7). Active group (24.1 ± 5.2)	Having periodontal disease. The term "periodontitis" was used to describe attachment loss of 3 mm on 3 teeth. 757 people have periodontal disease	Pregnant women between 6 and 20 weeks of gestation	Preterm labor	The goal of preventing preterm birth is not supported by screening for and treating periodontal disease during pregnancy. Active periodontal therapy during pregnancy might make various subtypes of preterm delivery more likely
Meqa et al. [6], Republic of Kosovo	2017	n = 200. Ages between 17 and 35 years old	Having periodontal disease. To determine whether the participants had periodontitis or not, Machtei's criteria were applied. The individuals in the periodontitis group had clinical attachment levels of at least 6 mm in two or more sites and periodontal pocket depths of at least 5 mm in one or more bleeding-positive sites. 50 patients have periodontitis overall	Two groups: before the 37th week of gestation and after the 37th week of gestation	Preterm birth and low birth weight	Periodontal disease increases the risk of delivering prematurely and a low birth weight child in pregnant women
Caneiro-Queija et al. [18], Spain	2019	n = 40. Ages between 18 and 40 years old. Control group (32.25 ± 4.21). Test group (32.14 ± 4.27)	Patients with acute periodontal disease. Clinical attachment level (CAL) and bleeding on probing (BOP) (six sites per tooth)	Women who are pregnant and have a gestational age of less than or equal to 16 weeks	Preterm birth and low birth weight	Non-surgical periodontal therapy did not significantly reduce the risk of adverse pregnancy outcomes in Caucasian patients with periodontitis second-stage grade B
Merchant et al. [19], United States	2018	n = 823. At Least 16 years old. Control group (25.9). Treatment group (26.1)	Had periodontal disease and they are equal to 823	Pregnancy with a singleton at less than 16 weeks and 6 days gestation	Preterm birth, spontaneous abortion, and stillbirth	Non-surgical periodontal treatment, such as scaling and root planning, given to mothers with mild to moderate periodontal disease before 21 weeks of gestation may help prevent preterm births
Jiang et al. [20], China	2016	n = 466. At least 30 years old	Exhibiting periodontal disease. Periodontal disease was determined to be present in participants with at least one sextant code that was equal to or higher than Code 3. Severe periodontal disease was considered to exist in females with at least one Code 4 region. 466 people had periodontal disease	Pregnant women with less than 20 weeks of gestation	Preterm birth and low birth weight, stillbirth, gestational diabetes mellitus, gestational hypertension, small for gestational age, severe anemia, preterm rupture of membranes, and fetal congenital anomalies	When pregnant women with periodontal disease, mouth rinses help to improve their periodontal problems. It is safe to use mouth rinse interventions when pregnant. While mouthwash had no impact on gestational age or birth weight, it greatly decreased the likelihood of an early membrane rupture
Jiang et al. [21], China	2015	n = 468. At least 18 years old	Periodontal disease will be identified in participants with at least one code of any sextant equal to or above three. There are 468 people who have periodontal disease	Pregnant women with less than 20 weeks gestation pregnant ladies	Preterm birth and low birth weight. Neonatal and infant mortality	The study's constraints (study period, research funding, and the study's relatively small sample size) hinder the proportional monitoring of birth outcomes
Fiorini et al. [22], Brazil	2013	n = 60. Ages between 18 and 35 years of age	49.62% of locations have been probed deeply. Third teeth are not allowed. Periodontal disease patients average 60 years old	Pregnant women with a gestational age of up to 20 weeks	Preterm birth and low birth weight	Reduced cytokine levels in pregnant women's gingival crevicular fluid were achieved with periodontal therapy. Systemic biomarkers of inflammation were unaffected by periodontal treatment. These findings might help to explain why periodontal therapy has not been able to reduce the frequency of preterm delivery, at least in part
Jiang et al. [23], China	2013	n = 470. At least 18 years old. Control group (26.0 ± 6.8). Treatment group (23.75 ± 4.85)	Any locations showing periodontal disease > 3 mm or CAL > 3 mm were considered to have periodontal disease	Pregnant women in 3rd trimester (32-35 gestational weeks). Women who are trying to get pregnant and want to do so within a year	Preterm birth and low birth weight	The study's constraints (study period, research funding, and the study's relatively small sample size) hinder the proportional monitoring of birth outcomes
Pirie et al. [24], Ireland	2013	n = 99. At least 18 years old. Control group (30.5 ± 5.5). Test group (30.5 ±4.5)	Having periodontal disease	Prior to week 22 of gestation, during the first trimester of pregnancy	Gestational age, birth weight, birth length, head circumference, and Apgar scores	In the participant population, non-surgical periodontal therapy administered between weeks 20 and 24 of pregnancy did not reduce the incidence of preterm and low-birth-weight deliveries
Weidlich et al. [25], Brazil	2012	n = 303. Ages between 18 and 35 years old	Plaque index, gingival index, supra-gingival calculus, cavities, overhanging restorations, bleeding on probing (BOP), periodontal probing depth (PPD), and clinical attachment level (CAL) were recorded	Pregnant women with a gestational age of 20 weeks or less. Singleton pregnancy	Preterm labor and low birth weight	Periodontitis in pregnant women can be successfully treated, which helps the condition. The decrease in preterm and/or low birth weight rates is not significantly impacted by the treatment of periodontitis in pregnant women
Oliveira et al. [26], Brazil	2011	n = 246. Ages between 18 and 35 years old. Intervention group (29.96 ± 4.38). Control group (26.58 ± 3.96)	Having periodontal disease. Clinical attachment level (CAL) and bleeding on probing (BOP) measurements were performed at six sites per tooth	Pregnant at a gestational age between 12 and 20 weeks	Preterm birth and low birth weight and pre-term low birth weight	The risk of preterm birth, low birth weight, and preterm low birth weight was not significantly decreased by periodontal therapy during the second trimester of pregnancy
Sant’Ana et al. [27], Brazil	2011	n = 33. Ages between 16 and 39 years old. Control group (26.0 ± 6.8). Treatment group (23.75 ± 4.85)	Pocket probing depth, clinical attachment level, sulcus bleeding index, and plaque index were recorded from four sites per tooth (mesiobuccal, buccal, distobuccal, and lingual) with a 15 mm periodontal probe, except for third molars. Patients with periodontal disease equal 33	Singleton gestations between 9 and 24 weeks	Preterm birth and low birth weight and abortion	Periodontal therapy administered during the second trimester of pregnancy helps to reduce unfavorable pregnancy outcomes
Jeffcoat et al. [28], United States	2010	n = 322. At least 18 years old. Control group and test group (23.7)	Having periodontal disease	Pregnant women at 6 to 20 weeks of gestation	Preterm birth and full-term birth	In the population analyzed in this experiment, effective routine periodontal therapy (scaling and root planning with oral hygiene teaching) is linked to a lower risk of spontaneous preterm birth
Offenbacher et al. [29], United States	2009	n = 1,806. At least 18 years old. Control group (25.4 ± 5.5). Treatment group (25.3 ± 5.5)	Having periodontal disease. At least 3 mm of clinical attachment loss	Pregnant women	Preterm labor hospitalization, pre-term birth, birth with maternal or fetal indications, triage, stillbirth, or spontaneous abortion, neonatal death, and congenital anomaly	Preterm birth rates were not decreased by periodontal therapy when a woman was pregnant
Herrera et al. [30], Colombia	2009	n = 60. At least 18 years old. Control group (27 ± 7.6). Intervention group (24 ± 6.5)	Chronic periodontitis	Pregnant women diagnosed with mild preeclampsia and gestational age between 26 and 34 weeks	Preeclampsia, eclampsia, HELLP ´s syndrome, low birth weight, pre-term birth, intrauterine growth restriction, and gestational diabetes	In participants with moderate preeclampsia, periodontal intervention does not appear to be harmful to health, to the severity of, or to change the frequency of maternal problems
Radnai et al. [31], Hungary	2009	n = 83. Ages between 16.2 and 43.1 years old. Control group (28.9 ± 5.4). Treatment group (29.1 ± 6)	Subjects are considered to have periodontitis if they had ≥ 4 mm probing depth, at least at one site, and bleeding on probing (BOP) for ≥ 50% of teeth. Participants having no ≥ 4 mm pockets or BOP occurring at less than 50% of teeth were regarded as periodontally healthy. Initial localized chronic periodontitis was found in 89 women	Singleton pregnancy	Preterm delivery and low birth weight	The likelihood of an unfavorable pregnancy outcome can be decreased if moms with chronic localized periodontitis receive periodontal care before the 35th gestational week
Newnham et al. [32], Australia	2009	n = 1,082. At least 16 years old	Having periodontal disease. Periodontal disease was defined as the presence of periodontal pockets of 4 mm or greater in depth at 12 or more probing sites in fully erupted teeth (typically excluding wisdom teeth). Periodontal pocketing was used to define the presence of periodontal disease rather than loss of clinical attachment. Patients with periodontal disease equal 1,082	Singleton pregnancy at more than 12 and less than 20 weeks of gestational age. Did not have any known fetal anomalies or other risk factors, such as hydramnios, that would place the pregnancy at imminent risk of complications	Preterm birth and low birth weight, preeclampsia, stillbirth, neonatal death, the onset of labor, mode of delivery, fever, retained placenta, primary postpartum hemorrhage, secondary postpartum hemorrhage, fetal and neonatal well-being (fetal heart anomalies and umbilical artery blood gas), neonatal head circumference, neonate length, and neonatal sepsis necessitating antibiotics	Treatment of periodontal disease in mid-pregnancy does not avert preterm birth, fetal growth restriction, or preeclampsia. A possible beneficial effect in preventing a proportion of stillbirths remains uncertain
Novak et al. [33], United States	2009	n = 83. Control group (25.7). Treatment group (27.3)	Having periodontal disease. Periodontitis defines if there was a pocket with a probing depth of 64 mm at least found at 1 site, and BOP at 150% of the dental sites. Patients with periodontal disease equal 83	Primigravida-primiparous, singleton, and healthy pregnancies	Preterm delivery, birth weight, 5-minute Apgar score, umbilical cord vein pH, and rate of prematurity	The study has suggested an association between the periodontal status of mothers and preterm delivery. Oral hygiene instructions to pregnant women with or without periodontitis were effective in reducing the rate of preterm delivery and inducing a higher birth weight and consequently a better neonatal outcome
Michalowicz et al. [34], United States	2007	n = 823. At least 16 years old. Control group (25.9 ± 5.5). Treatment group (26.1 ± 5.6)	Having periodontal disease. Periodontal disease is defined as 4 or more teeth with a probing depth of at least 4 mm and a clinical attachment loss of at least 2 mm, as well as bleeding on probing at 35% or more of tooth sites. Patients with periodontal disease equal 823	Singleton pregnancy at less than 16 weeks and 6 days of gestational age	Preterm labor, low birth weight, birth length, preeclampsia, Apgar score, and admission to the neonatal intensive care unit	Treatment of periodontitis in pregnant women is safe and improves periodontal disease. Treatment of periodontitis in pregnant women does not significantly alter rates of preterm birth, low birth weight, or fetal growth restriction
Tarannum and Faizuddin [35], India	2007	n = 200. Ages between 18 and 35 years old. Control group (22.9 ± 3.6). Treatment group (23 ± 3.3)	Having periodontal disease. Patients who experienced an attachment loss of at least 2 mm at 50% or more of the sites under examination are included	Healthy pregnant women with gestational age between 9 and 21 weeks, with a singleton fetus	Preterm birth and low birth weight	Prenatal periodontal care can lower the likelihood of preterm birth
Sadatmansouri et al. [36], Iran	2006	n = 30. Ages between 18 and 35 years old. Control group (28.4 + 4.1). Test group (29.1 + 4.3)	Having a periodontal disease that is mild or advanced. Investigated were clinical attachment loss and hemorrhage upon probing. The distance between the gingival edge and the base of the pocket	Pregnant at 13 to 20 weeks of gestation	Preterm low birth weight	Periodontal treatment phase 1 results in a reduction in preterm low birth weight
López et al. [37], Chile	2005	n = 870. Ages between 18 and 42 years old. Control group (24.8 ± 2.7). Treatment group (25 ± 2.9)	A dental examination revealed that they had periodontal disease. On the six sites, bleeding on probing (BOP) was evaluated. The gingival redness and the lingual or buccal tooth were recognized	Pregnant women with gestational age between 9 and 21 weeks. Singleton gestation	Preterm birth and low birth weight	An independent risk factor for preterm low birth weight appears to be periodontal disease. When utilized in a population of women with periodontal disease, periodontal treatment significantly reduced the odds of preterm low birth weight
López et al. [38], Chile	2002	n = 400. Ages between 18 and 35 years old. Treatment group (28 ± 4.5). Control group (27 ± 4.3)	Having periodontal disease. On the six sites, bleeding on probing was evaluated. Determined were the lingual or buccal tooth and gingival redness	Pregnant women with gestational age between 9 and 21 weeks with a singleton fetus	Preterm birth and low birth weight	Periodontal disease appears to be a separate risk factor for preterm low birth weight, with a four-fold increase in the likelihood of preterm low birth weight. The rates of preterm low birth weight have been significantly decreased by periodontal therapy when used in such a population of women having periodontal disease

Primary Outcomes

The primary outcomes demonstrated the number of studies that reported a significant association between periodontal diseases and adverse pregnancy outcomes. Three studies out of 23 focused mainly on the relationship between periodontal disease and adverse pregnancy outcomes [6,33,38]. All three articles showed a significant association between acute/chronic periodontal diseases and adverse pregnancy outcomes [6,33,38]. An informative summary of the article’s features and their main outcomes is illustrated in Table 1.

Secondary Outcomes

The secondary outcomes described the impact of periodontal diseases on adverse pregnancy outcomes such as low birth weight, preterm birth, preeclampsia, eclampsia, HELLP syndrome, intrauterine growth restriction, and gestational diabetes. All studies focused on and observed the relationship between periodontitis and both preterm delivery and low birth weight [3,6,18-38]. Moreover, six studies observed neonatal mortality such as stillbirth and abortion in addition to preterm delivery and low birth weight [19-21,27,29,32]. Table 1 illustrated the types of adverse pregnancy outcomes observed through all included studies. In addition, secondary outcomes demonstrated the number of studies that reported a significant impact of periodontal disease treatment on reducing the risk of adverse pregnancy outcomes. Twenty-three articles focused on and observed the impact of periodontal disease on reducing the risk of adverse pregnancy outcomes [3,6,18-38]. Out of 23 studies, 11 showed a significant association and positive impact of periodontitis therapy on decreasing the risk of adverse pregnancy outcomes [6,19,25,27,28,31,33,35-38]. In contrast, nine studies showed no impact of periodontal disease therapy on reducing adverse pregnancy outcomes [3,18,22,24,26,29,30,32,34]. One study showed a positive impact of periodontitis treatment on some adverse pregnancy outcomes and no impact on other adverse pregnancy outcomes [20]. In addition, two studies showed limitations in assessing the impact of periodontal disease treatment on reducing adverse pregnancy outcomes because of the small size of the sample in both studies [21,23]. An informative summary of the article’s features and their main outcomes is illustrated in Table 1.

Quality and Risk Assessment of the Included Articles

**Figure 2:**
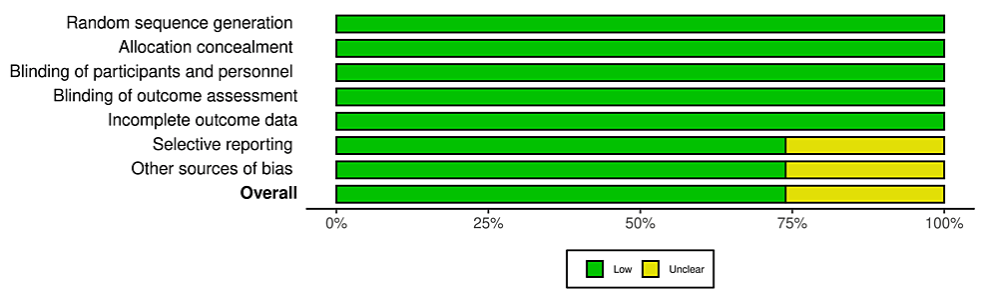
The overall risk of bias.

**Figure 3:**
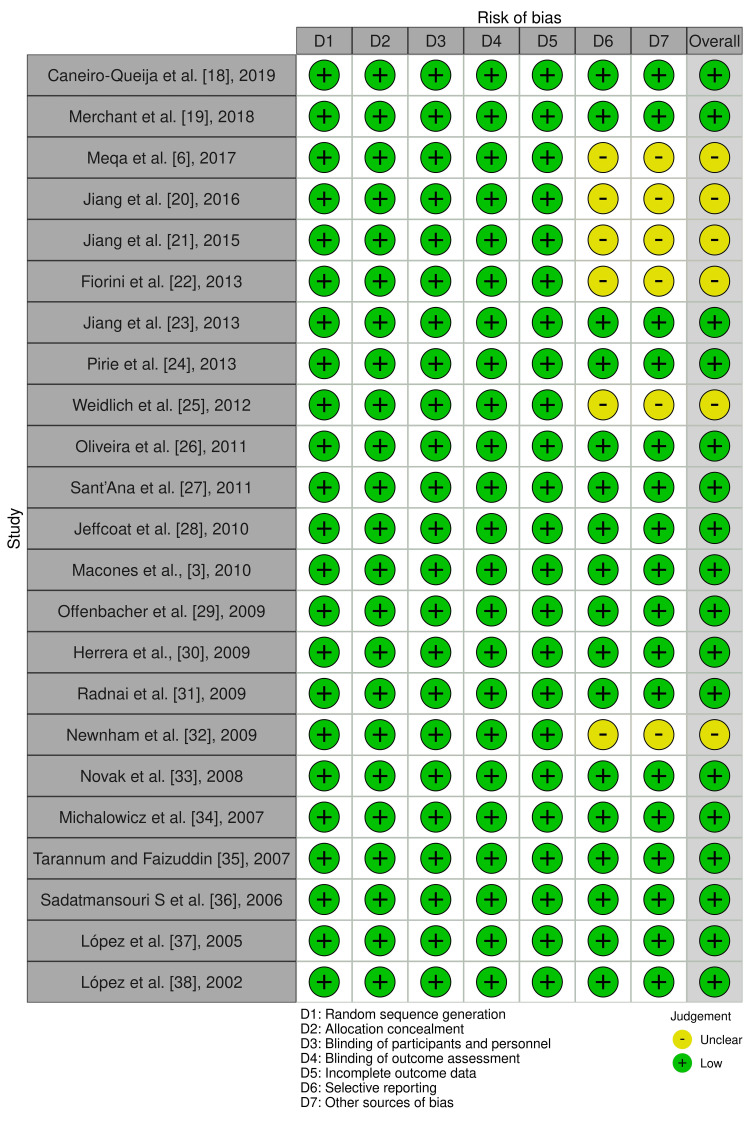
The risk of bias assessment of each study.

The quality and risk assessment of all included articles were concluded by four authors. All articles that have been included were determined by following the Cochrane risk-of-bias visualization (robvis) [17] to assess the risk of bias. The preponderance of the included articles had a low risk of bias in the following domains: selective reporting (73.9%) and other sources of bias (73.9%). All studies revealed a low risk of bias (100%) in random sequence generation, allocation concealment, blinding of participants and personnel, blinding of outcomes assessment, and incomplete outcome data domains (Figure 2). Thoroughly, among the 23 studies, only six studies (26.1%) had an unclear risk of bias. Seventeen studies (73.9%) had a low risk of bias as demonstrated in Figures 2, 3. The scoring of unclear risk of bias was given to six studies due to inadequacy of sufficient information to make a clear judgment in the following domains: selective reporting and other sources of bias (Figure 3).

Discussion

This systematic review aimed to evaluate and illustrate research findings that fulfilled this research objective. All randomized control clinical trials that studied the relationship between periodontitis and adverse pregnancy outcomes and the impact of periodontal therapy on adverse pregnancy outcomes have been included, with no time restriction. Our review exhibited an exhaustive group of evidence collected from 23 studies that satisfied the inclusion and exclusion criteria.

The primary outcome of this systematic review is to assess the association between periodontitis and adverse pregnancy outcomes. All the studies that are included in this review and focused on this objective reported a significant association between periodontitis and adverse pregnancy outcomes [6,33,38]. In contrast, research failed to catch periodontal bacteria in the amniotic fluid of women with periodontitis who delivered preterm, despite these pathogens being often found in dental plaque. In addition, *Fusobacterium nucleatum* in vaginal swab samples and dental plaque were not associated and related to the presence of the bacteria in amniotic fluid [36].

The secondary outcomes reported the impact of periodontal disease therapy in reducing the influence of adverse pregnancy outcomes. The effect varied among studies in which several studies reported a significant association between periodontitis therapy and decreasing the risk of adverse pregnancy outcomes in pregnant women with periodontitis. Merchant et al. [19] disclosed that periodontal treatment may prevent preterm births. This therapy was provided to mothers at a gestational age of less than 21 weeks with mild to moderate periodontal disease. The treatment group had 49 preterm births in comparison to the control group, which had 52 preterm births, and five spontaneous abortions or stillbirths in the treatment group in comparison to 14 spontaneous abortions or stillbirths in the control group. This explains the favorability of using periodontal therapy to decrease the risk of adverse pregnancy outcomes such as preterm births and spontaneous abortions or stillbirths. The intention-to-treat hazard ratio for preterm birth comparing the treatment versus control groups was 0.93 (95% CI: 0.63-1.37). On the other hand, Pirie et al. [24] reported that non-surgical periodontal treatment at a gestational age between 20 and 24 weeks in the white population did not reduce adverse pregnancy outcomes including preterm and low birth weight. This was justified by the fact that the differences are insignificant between both the control and test groups in regard to birth length (P = 0.64), Apgar scores, type of delivery, and head circumference (P = 0.29). Moreover, both groups had similar cytokine levels in cord serum. Pirie et al. [24] mentioned that the difference is insignificant between the control and test groups in terms of gestational age (P = 0.23) and birth weight (P = 0.89). Results show that gestational age was slightly lower, and the birth weight was higher in the test group. Another randomized control trial performed by Jiang et al. [20] disclosed that periodontal therapy has a significant impact in decreasing the rate of premature rupture of the membranes but has no effect on gestational age or birth weight. They used alcohol-free antimicrobial cetylpyridinium chloride (CPC) mouth rinse during pregnancy. The women in the treatment group had a significantly lesser rate of premature rupture of the membranes (1.4%) in comparison with the control group (5.7%) (OR = 0.23, 95% CI = 0.07-0.84, P = 0.03).

Up to date, there are six systematic reviews that assessed the relationship between periodontitis and adverse pregnancy outcomes [2,39-43], and only 10 systematic reviews assessed the impact of periodontal treatment on decreasing the risk of adverse pregnancy outcomes in all the past years [4,43-51]. Of these systematic reviews, five systematic reviews supported the association between periodontal diseases and adverse pregnancy outcomes [2,39-42]. In contrast, only one systematic review reported no association between periodontitis and adverse pregnancy outcomes [43]. In addition, four systematic reviews supported periodontal therapy in reducing the influence of adverse pregnancy outcomes [45,48,50,51], and four systematic reviews concluded periodontal disease therapy has no effect on decreasing the risk of adverse pregnancy outcomes [4,46,47,49]. Furthermore, one systematic review reported that periodontal therapy has a positive impact on reducing the risk of low birth weight, but no difference was seen in preterm birth, small for gestational age, preeclampsia, or perinatal mortality [43]. Additionally, one systematic review had insufficient differential findings to assess the impact of periodontal therapy on adverse pregnancy outcomes [43].

Pockpa et al. [2] did a review of clinical studies over two decades and published it in 2021. This review included 232 articles (n = 119,774 participants). Geographical interpretation declared that most of the included studies were conducted in the United States (42 studies, 18.10%), followed by Brazil (33 studies, 14.22%) and then India (25 studies, 10.78%) [2]. The preponderance of the studies reports a statistically significant association between periodontitis and preterm birth (63.96%), low birth weight (71.87%), preterm low birth weight (59.18%), preeclampsia (68.89%), and other pregnancy complications like premature rupture of membrane (65.38%) [2]. Moreover, Iheozor-Ejiofor et al. in 2017 did a review to assess the impact of periodontal disease treatment in pregnant women to decrease perinatal morbidity and fatality [44]. Fifteen randomized control trials were included in this review (n = 7161 participants). Periodontal treatment with the control group during pregnancy has been compared in 11 studies and the meta-analysis reported no difference in regard to preterm birth (RR: 0.87, 95% CI: 0.70 to 1.10; 5671 participants; 11 studies; low-quality evidence). Also, it showed that periodontal treatment may decrease low birth weight < 2500 g (9.70% with periodontal treatment versus 12.60% without treatment; RR: 0.67, 95% CI: 0.48 to 0.95; 3470 participants; seven studies; low-quality evidence) [44]. It is ambiguous if periodontal therapy has an effect on reducing perinatal mortality (RR: 0.85, 95% CI: 0.51 to 1.43; 5320 participants; seven studies; very low-quality evidence) and preeclampsia (RR: 1.10, 95% CI: 0.74 to 1.62; 2946 participants; three studies; very low-quality evidence). There is no evidence of a difference in small for gestational age (RR: 0.97, 95% CI: 0.81 to 1.16; 3610 participants; three studies; low-quality evidence) [44]. It concluded that it is ambiguous with low-quality evidence if periodontal therapy during pregnancy has an effect on preterm birth, and there is also low-quality evidence that periodontal therapy may reduce low birth weight [44].

Obstetric physicians should take this association into consideration by guiding and advising any pregnant woman complaining of periodontitis symptoms to visit the dentist to treat it. Treatment of periodontitis is very simple and cost-effective in comparison with managing adverse pregnancy outcomes. This approach will help in decreasing the incidence of adverse pregnancy outcomes and in cutting costs. Furthermore, preconception screening for periodontitis to treat it before conception and oral hygiene instruction will play a very important role in eliminating periodontitis as a risk factor. Moreover, periodic dental visits during pregnancy should be part of the antenatal visits and assessments to detect periodontitis in its mild stage and treat it.

In summary, this systematic review illustrated an overview of different articles exhibiting the relationship between periodontal diseases and adverse pregnancy outcomes as well as the effect of periodontal disease therapy on adverse pregnancy outcomes (Table 1). It can be established that there are deficient data to relieve the relationship between periodontal diseases and adverse pregnancy outcomes, and the effectiveness of periodontal treatment as a reducing factor of adverse pregnancy outcomes to treat pregnant women with periodontal diseases for now. However, the results from the included studies in this review advocate the need for more clinical trials to be capable to acknowledge periodontal therapy as a reducing factor of adverse pregnancy outcomes in pregnant women with periodontitis to be an approach in these types of patients. Obstetric physicians should take this association into consideration to decrease the incidence of adverse pregnancy outcomes by guiding these kinds of patients to the dentist to treat periodontitis. Moreover, medical and dental clinical assessment in pregnant women is essential before utilizing this form of therapy in pregnant women with periodontal diseases. The strength of this current review is that it included 23 articles that have been peer-reviewed and published from 2002 to 2019.

Study Strengths and Limitations of This Systematic Review

Our systematic review accumulated and assessed all peer-reviewed studies published in the past years that met the inclusion criteria. We used Scopus, PubMed, Google Scholar, and Web of Science as search instruments. One superiority of utilizing Google Scholar is that it averts researchers from missing any important study that has been published in journals but has not yet been cited in Scopus, Web of Science, or PubMed. To avoid making our question too specific, we used broad and general search terms. Furthermore, studies included were conducted on human subjects only and only randomized controlled trials were included. On the other hand, meta-analysis was impossible because of the discrepancy in the included articles. Moreover, future systematic reviews should include different study designs such as observational studies to evaluate the relationship between periodontal diseases and perinatal morbidity and mortality, malformations, and chromosomal abnormalities.

## Conclusions

This systematic review revealed that there is an association between periodontal diseases and adverse pregnancy outcomes, and periodontal therapy has a reducing effect on adverse pregnancy outcomes in pregnant women with periodontitis. Obstetric physicians and dentists should take this association into consideration to treat periodontitis in pregnant women to decrease the incidence of adverse pregnancy outcomes and cut costs. Future studies are warranted to investigate the relationship between periodontitis and different adverse pregnancy outcomes and to decide the best type and the most effective therapy to treat periodontitis in pregnant women.
